# Induction of Apoptosis in Human Breast Adenocarcinoma Cells MCF-7 by Monapurpyridine A, a New Azaphilone Derivative from *Monascus purpureus* NTU 568

**DOI:** 10.3390/molecules17010664

**Published:** 2012-01-11

**Authors:** Li-Chuan Hsu, Ya-Wen Hsu, Yu-Han Liang, Chia-Ching Liaw, Yao-Haur Kuo, Tzu-Ming Pan

**Affiliations:** 1 Department of Biochemical Science and Technology, National Taiwan University, Taipei 10617, Taiwan; 2 Division of Herbal Drugs and Natural Products, National Research Institute of Chinese Medicine, Taipei 11221, Taiwan; 3 Graduate Institute of Integrated Medicine, China Medical University, Taichung 40402, Taiwan

**Keywords:** *Monascus purpureus* NTU 568, azaphilone, monapurpyridine A, cytotoxicity, apoptosis

## Abstract

A new azaphilonidal derivative, monapurpyridine A (**MPA**), has recently been isolated from the fermented products of *Monascus purpureus* NTU 568. The structure of **MPA** was elucidated by nuclear magnetic resonance (^1^H-NMR, ^13^C-NMR, COSY, HMQC, and HMBC) and other spectroscopic analyses. Biological evaluation revealed that **MPA** could induce cell death in human breast adenocarcinoma cells MCF-7, and it has no significant toxicity to normal mammary epithelial cells M10. The MTT assay and flow cytometric analysis were employed to investigate cell viability and cell cycle influenced by **MPA**. Moreover, we used Western blot and caspase activity assay to demonstrate the activation of caspase-3, -8 and -9 resulted from **MPA**. All evidence supported that **MPA** was suitable for developing into a chemotherapeutic or chemopreventive agent against breast cancer.

## 1. Introduction

*Monascus* species have traditionally been used as food additives in Asian countries for thousands of years. Recently, *Monascus*-fermented rice, also called red mold rice (RMR), has been reported to possess various biological functions, such as: hypolipidemic effects [1], antifatigue activities [2], neuroprotective properties against Alzheimer’s disease [3], preventive ability for obesity [4], and prevention of carcinogenesis [5] or tumor progression [6], *etc*.

Some bioactive secondary metabolites from *Monascus* species have been identified and their biological activities proven. For example, monacolin K is a kind of HMG-CoA reductase inhibitor [7], γ-aminobutyric acid (GABA) could reduce hypotension [8,9], and dimerumic acid was an anti-oxidant [10]. In addition, yellow pigments possessed anti-tumor and anti-inflammatory effects [11,12,13].

Recently, a variety of new azaphilones were isolated and characterized from *Monascus*-fermented products. For example, monapurones A–C were isolated from the extract of RMR and showed selective cytotoxicity against human lung cancer cell line A549, while exhibiting no significant toxicity to human normal lung cells MRC-5 and WI-38 [14]. Four new pyridine derivatives, monasnicotinates A–D were isolated from *Monascus pilosus* BCRC 38093 and evaluated for their inhibitory effects against lipopolysaccharide (LPS)-induced nitric oxide production [15]. 

**Figure 1:**
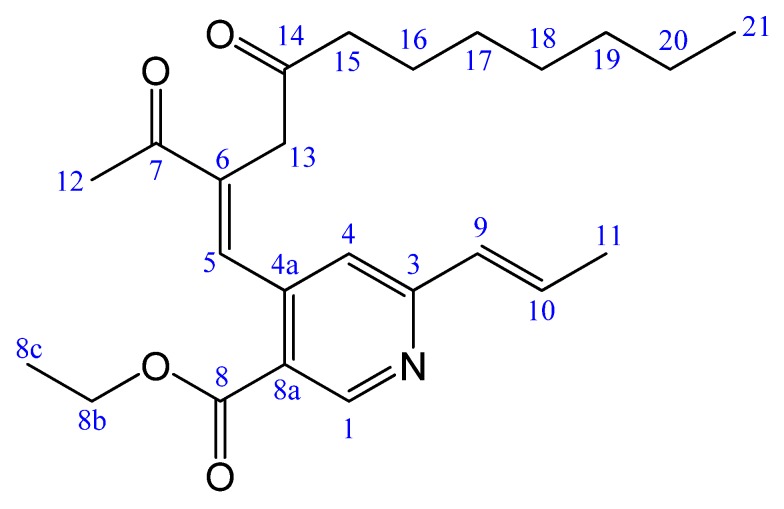
The structure of monapurpyridine A.

In our laboratory, five new azaphilone pigments, including two blue fluorescent monapurfluores have been isolated from *Monascus purpureus* NTU 568. These new azaphilones were reported to be cytotoxic to cancer cell lines or anti-inflammatory on LPS-stimulated Raw 264.7 cells [16,17]. We also executed a large-scale preparation for monaphilone A, one of these new azaphilones, to explore the apoptosis-related and anti-inflammatory properties in inducing death of human laryngeal carcinoma cell line HEp-2 and reducing inflammatory responses on RAW 264.7 cells [18]. Here, we report the structural elucidation of the new isolated compound, monapurpyridine A (**MPA**; Figure 1), and its apoptosis-related mechanisms. For these purposes, we obtained **MPA** on a large scale and designed some experiments to induce apoptosis in the human breast cancer cell line MCF-7.

## 2. Results and Discussion

### 2.1. Structure Determination

**Figure 2:**
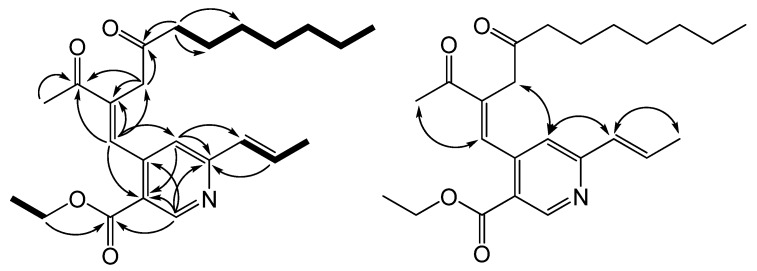
Key ^1^H-^1^H COSY (▬), HMBC (H→C) and NOESY (H↔H) correlations of **MPA**.

Monapurpyridine A (**MPA**) was obtained as a yellow oil. The HRESIMS of **MPA** showed a molecular ion at *m/z* 400.2480 ([M+H]^+^, C_24_H_34_NO_4_), indicating a molecular formula of C_2__4_H_33_NO_4_ (calcd. 399.2410), which contains nine required degrees of unsaturation. The IR spectrum showed bands at 1,716 and 1,675 cm^−1^, consistent with the presence of conjugated ketone and carboxylic ester groups. The UV maximum at 261 and 308 nm inferred pyridine-chromophore system. The ^1^H and ^13^C spectra (Table 1) of **MPA** disclosed the signals for the presence of ketones (*δ*_C_ 208.7, C-14; 198.8, C-7), ester carbonyl (*δ*_C_ 165.2, C-8), trisubstituted pyridine unit (*δ*_C_ 152.0, 159.4, 120.1, 145.5, and 121.4; *δ*_H_ 9.11, s, H-1; 7.21, s, H-4), trisubstituted olefinic unit (*δ*_C_ 141.1, 136.9; *δ*_H_ 8.09, s, H-5), *trans*-double bond (130.4, 135.1; *δ*_H_ 6.47, d, *J* = 15.6; 6.85, d, *J* = 15.6, 6.8), one ethyl group (*δ*_H_ 4.34, 2H, *J* = 6.4; 1.37, 3H, *J* = 6.4; *δ*_C_ 61.4, 14.3), seven methylene carbons (*δ*_C_ 40.7, 43.0, 23.8, 29.1, 29.1, 31.6, 22.6), as well as three methyls (*δ*_H_ 2.48, s; 1.92, d, *J* = 6.8; 0.84, t, *J* = 7.2). As shown in Figure 2, the correlations of three partial structures (H_2_-8b/Me-8c, H-9/H-10/Me-11, and H_2_-15/H_2_-16/H_2_-17/H_2_-18/H_2_-19/H_2_-20/Me-21) were confirmed by analysis of ^1^H-^1^H COSY (the bold) and HMBC (the arrows) correlations. From the HMBC spectrum, the methylene protons at *δ*_H_ 3.25 (H_2_-13) were correlated between trisubstituted olefinic unit and two ketone carbonyls, together with the correlations of H_2_-15/C-14 and Me-12/C-7, thus the (CH_2_)_6_CH_3_ group and Me-12 (*δ*_H_ 2.48) were determined at C-14 and C-7, respectively. Furthermore, the key HMBC correlations between H-1/C-3, C-4a, C-8a, H-4/C-3, C-8a, C-9, H-5/C-4, C-7, C-6, C-13, H_2_-8b/C-8 revealed that the fragments were located at C-3, C-4a and C-8a positions of trisubstituted pyridine unit. Thus, the planer structure of **MPA** was completely assigned by 2D NMR experiments, especially ^1^H-^1^H COSY, HMQC, and HMBC. The relative stereochemistry of **MPA** was further determined, due to the NOESY (Figure 2) spectrum showing the correlations between H-9/Me-11, H-4/H2-13, and H-5/Me-12 indicating *E*-forms of C-9/10 and C-5/6 double bonds. Based on the above findings, the **MPA** was established as ethyl 4-((*E*)-2-acetyl-4-oxoundec-1-enyl)-6-((*E*)-prop-1-enyl)nicotinate, and has been named monapurpyridine A.

**Table 1:** ^1^H-and ^13^C-NMR spectroscopic data of **MPA** (in CDCl_3_) ^a,b^.

No.	δ_Η_ (400 MHz, *J* in Hz)	δ_C_ (100 MHz)	No.	δ_Η_ (400 MHz, *J* in Hz)	δ_C_ (100 MHz)
1	9.11 (*s*)	152.0	10	6.85 (*dq*, *J* = 15.6, 6.8)	135.1
3		159.4	11	1.92 (*d*, *J* = 6.8, 3H)	18.6
4	7.21 (*s*)	120.1	12	2.48 (*s*, 3H)	25.4
4a		145.5	13	3.25 (*s*, 2H)	40.7
5	8.09 (*s*)	141.1	14		208.7
6		136.9	15	2.50 (*t*, *J* = 7.2, 2H)	43.0
7		198.8	16	1.55 (*m*, *J* = 7.2, 2H)	23.8
8		165.2	17	1.28 (*m*, 2H)	29.1
8a		121.4	18	1.28 (*m*, 2H)	29.1
8b	4.34 (*q*, *J* = 6.4, 2H)	61.4	19	1.28 (*m*, 2H)	31.6
8c	1.37 (*t*, *J* = 6.4, 3H)	14.3	20	1.28 (*m*, 2H)	22.6
9	6.47 (*d*, *J* = 15.6)	130.4	21	0.84 (*t*, *J* = 7.2, 3H)	14.0

^a^ Assignments were confirmed by ^1^H-^1^H COSY, HMQC, HMBC; ^b^ *m*: multiple signal.

### 2.2. Cytotoxicity of MPA on MCF-7 and M10 Cells

**Figure 3:**
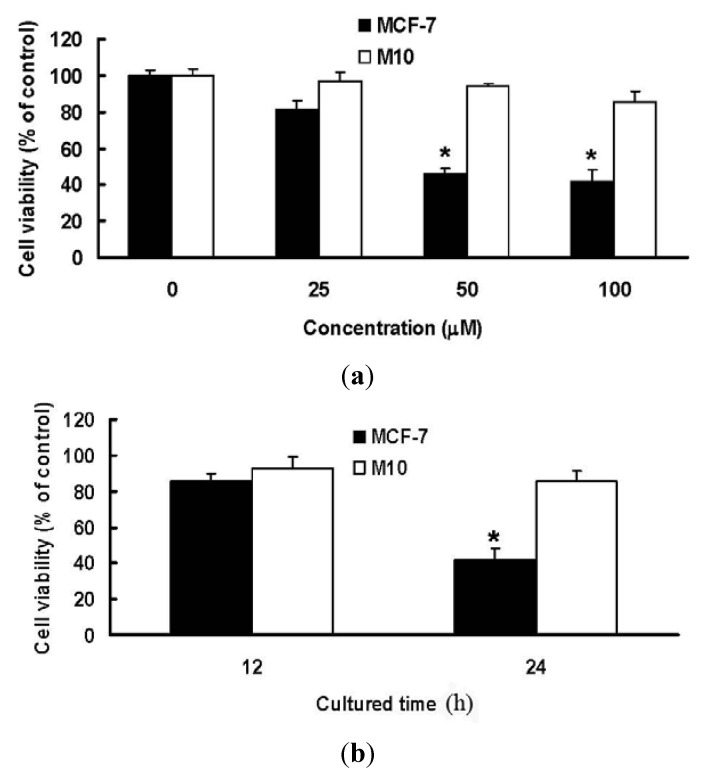
The effects of MPA on cell viability of MCF-7 and M10 cells. (**a**) Cells were treated with 25, 50 and 100 μM MPA for 24 h; (**b**) Cells were treated with 100 μM MPA for 12 and 24 h. Data were expressed as means ± SD (*n* = 3). * Significantly different (*p* < 0.01) *versus* the negative control (without any treatment).

We utilized MTT assay for a two-day course to study the inhibition on cell viability of MCF-7 and M10 cells treated with MPA (Figure 3). Up to the concentration of 100 μM, MPA showed dose-dependent and moderated cytotoxic activity against MCF-7 cells, but no significant cytotoxicity to normal M10 cells. The results suggested that MPA was selectively cytotoxic to breast cancer cell line.

### 2.3. Cell Death Induced by MPA on MCF-7 Cells

**Figure 4:**
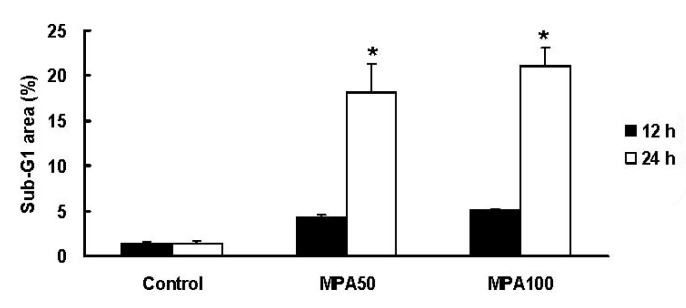
Flow cytometric analysis of Sub-G_1_ area of MCF-7 cells treated with MPA. MCF-7 cells were treated with 50 and 100 μM MPA for 12 and 24 h. Data were expressed as means ± SD (n = 3). * Significantly different (p < 0.05) *versus* the negative control (without any treatment).

To study cell deaths of MCF-7 cells induced by MPA, we utilized flow cytometry (propidium iodide staining) to analyze the ratio of Sub-G_1_ area for 12 and 24 h (Figure 4). MPA (50 μM, 24 h) significantly induced about 10-fold more cell deaths than control group. To make clear that the cell death resulted from apoptosis or necrosis, we designed some apoptotic approaches in the next step.

### 2.4. Caspase Activation of MPA on MCF-7 Cells

**Figure 5:**
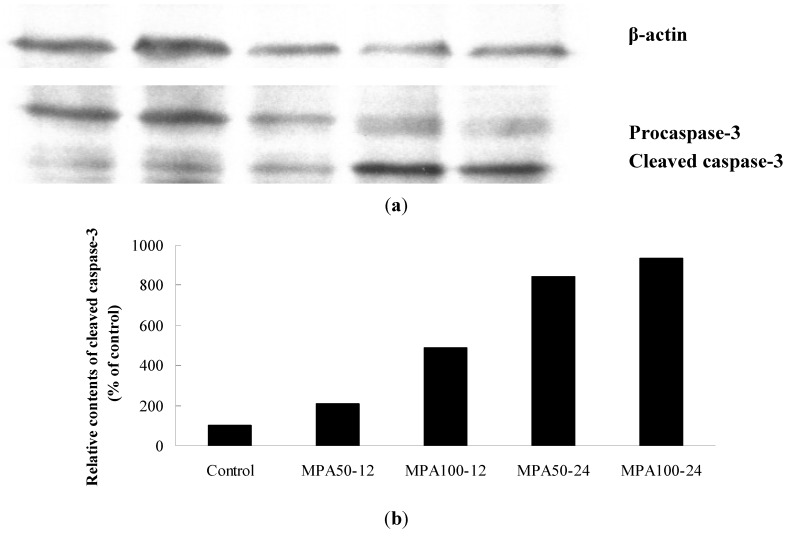
The effects of MPA on caspase-3 activation in MCF-7 cells. Cleaved caspase-3 and β-actin were detected by Western blot. MCF-7 cells were treated with 50 or 100 μM of MPA for 12 or 24 h. (**a**) From the left side: Lane 1, control; lane 2, MPA 50 μM for 12 h; lane 3, MPA 100 μM for 12 h; lane 4, MPA 50 μM for 24 h; lane 5, MPA 100 μM for 24 h. (**b**) Quantification of cleaved caspase-3 presented above.

**Figure 6:**
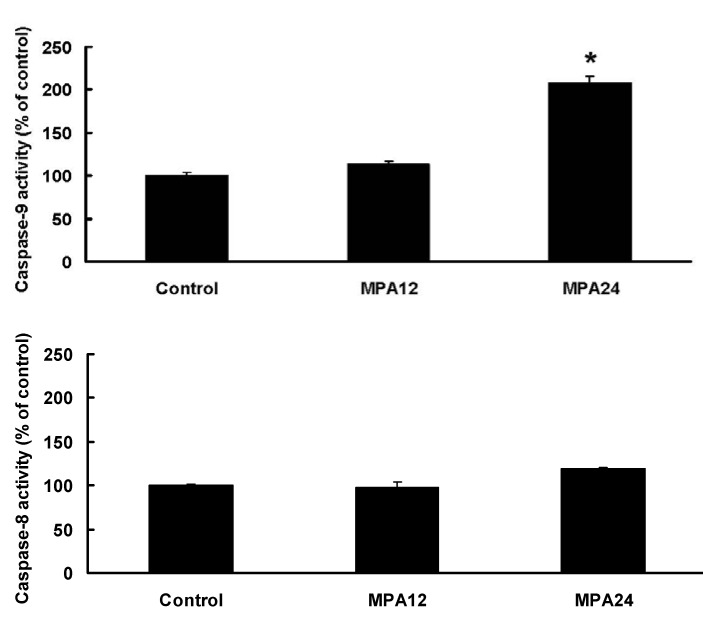
The effects of MPA on caspase-9 and -8 activities. MCF-7 cells were treated with 50 μM of test agents for 12 or 24 h. From the left side were: control; MPA, 12 h; MPA, 24 h. Data were expressed as means ± SD (*n* = 3). * Significantly different (*p* < 0.01) *versus* the control (without any treatment).

MCF-7 cells were treated with 50 and 100 μM of MPA for 12 and 24 h, and further analyzed for the cleaved caspase-3 by Western blot (Figure 5) and enzyme activity of caspase-8 and -9 by colorimetric assay kit (Figure 6). Treatment of MPA (50 and 100 μM, 24 h) exhibited increases of cleaved caspase-3, which were estimated as a down-streamed event of apoptosis. As to the up-streamed caspase-9 and caspase-8, treatment of MPA (50 μM, 24 h) exhibited significant increases of caspase-9 activity, but showed no significant increase of caspase-8. Thus, MPA was demonstrated to induce apoptosis through caspase-9 activations.

## 3. Experimental

### 3.1. General

Electrospray ionization mass spectrometry (ESI-MS) data were acquired on a LCQ mass spectrometer (Finnigan MAT LCQ, San Jose, CA , USA). NMR spectra were run on a Bruker Unity Plus 400 MHz NMR spectrometer (Bruker BioSpin, Rheinstetten, Germany) using CDCl_3_ as the solvent. Sephadex LH-20 (GE Healthcare, Uppsala, Sweden) and silica gel 60 (70–230 mesh and 230–400 mesh, Merck, Darmstadt, Germany) were used as chromatographic supports. Silica Gel 60 F254 plates (Merck) were used for thin layer chromatography (TLC). The TLC spots were detected under UV-lamps (254 and 365 nm) and also by using an anisaldehyde-sulphuric acid solution, applied as a spray reagent, followed by heating. The high performance liquid chromatography (HPLC) was performed using a Shimadzu LC-6AD apparatus with a SPD-6AV UV detector that was equipped with a preparative Cosmosil AR-II column (250 × 20 mm i.d., Nacalai Tesque, Inc., Kyoto, Japan).

### 3.2. Reagents

Methanol and acetonitrile (HPLC grade), acetone, ethyl acetate, *n*-hexane and methanol (analytical grade) were purchased from ECHO (Miaoli, Taiwan). Trifluroacetic acid (TFA), anisaldehyde and sulphyric acid were purchased from Merck. Fetal bovine serum (FBS), minimum essential medium (MEM), Dulbecco’s minimum essential medium (DMEM), phosphate buffered saline (PBS) and trypan blue were purchased from Biological Industries (Kibbutz Beit-Haemek, North District, Isreal). Other chemicals, such as 3-(4,5-dimethylthiazol-2-yl)-2,5-diphenyl-tetrazolium bromide (MTT), dimethyl sulfoxide (DMSO) and propidium iodide were obtained from Sigma (St. Louis, MO, USA).

### 3.3. Extraction and Isolation

The RMR powder (5 kg) was extracted with methanol (25 L) at 50 °C for 24 h. The dried extract was subjected to silica gel column chromatography, eluting with a mixture of *n*-hexane/ethyl acetate (9:1, 8:2, 7:3, 6:4, 0:10). This fraction (8:2) was then further separated by Sephadex (LH-20) gel column to remove other impurities and then purified again using preparative HPLC (Cosmosil 5C_18_ packing column, 250 × 20 mm i.d., MeOH/H_2_O = 85:15, 7 mL/min) to obtain MPA (6.6 mg).

### 3.4. Spectral Data

Monapurpyridine A (**MPA**): Yellow oily liquid. IR (neat) *ν*_max_ 1,716, 1,675, 1,588, 1,369, 1,284, 1,174, 1,094 cm^−1^. UV (MeOH) *λ*_max_ (log ε): 261 nm (3.52), 308 nm (3.01). HRESIMS *m/z* 400.2480 ([M+H]^+^, C_24_H_34_NO_4_). ^1^H-NMR and ^13^C-NMR data were listed in Table 1.

### 3.5. Cell Lines and Culture Conditions

Human breast adenocarcinoma cells MCF-7 and normal mammary epithelial cells M10 were obtained from Bioresources Collection and Research Center (Hsinchu, Taiwan). Both cell lines were maintained in MEM (5% FBS) in a humidified incubator with 5% CO_2_ at 37 °C.

### 3.6. Cytotoxicity Assay

Cells (3 × 10^3^ per well) were seeded with MEM (180 μL) in 96-well plates. After 4 h, test agents dissolved in PBS solution (20 μL) were added at final concentrations of 25, 50 and 100 μM and incubated in a 37 °C incubator with 5% CO_2_. After culturing for 24, 48 h, MTT solution (2 mg/mL, 20 μL) was added to each well and incubated for 4 h to induce the cellular conversion of the tetrazolium salt into a formazan product. The supernatant was then removed and DMSO (200 μL) was added to dissolve the formazan, which can be detected by spectrophotometry at 570 nm and provided a relative estimate of cell viability.

### 3.7. Assay of DNA Contents by Flow Cytometry

MCF-7 cells (5 × 10^4^ per well) were seeded with MEM (2 mL) in 6-well plates. After 12 h, test agents dissolved in MEM solution (2 mL) were added at final concentrations of 50 and 100 μM. After 12 and 24 h of incubation, the cells were harvested and fixed with 80% ethanol for 30 min. Then the cell pellets were washed three times with PBS and co-incubated the cells with propidium iodide (4 μg/mL), Triton X-100 (1%), and RNase (0.1 μg/mL) in the dark for 30 min. Finally, the cells can be analyzed by flow cytometry (FACSCalibur, Ser. No. E1577, BD) equipped with Cell Quest software to provide a relative estimate of DNA contents.

### 3.8. Western Blot Analysis

Cells (about 5 × 10^5^) were seeded with media (10 mL) in a 75 cm^2^ flask. After 12 h, test agents dissolved in media (10 mL) of were added. After 12 and 24 h of incubation, the cells were harvested and extracted by RIPA lysis buffer (Millipore, Bellerica, MA, USA) with 1% protease inhibitor (Sigma, St. Louis, MO, USA). The cell lysates were analyzed with primary antibodies, including of caspase-3 antibody (Novus Biologicals, Littleton, CO, USA) and β-actin antibody (Epitomics, Burlingame, CA, USA). The anti-mouse secondary horseradish peroxidase antibodies (Jackson ImmunoResearch, West Grove, PA, USA) was further added. Finally, the detection was performed using the Western lightning chemiluminescence reagent (PerkinElmer Life Sciences, Waltham, MA, USA).

### 3.9. Caspase Activity Assay

Cells (about 5 × 10^5^) were seeded with media (10 mL) of in a 75 cm^2^ cell culture flask. After 12 h, test agents dissolved in media (10 mL) were added. After 12 and 24 h of incubation, the cells were harvested and tested for caspase-8 and caspase-9 activities respectively using a colorimetric assay kit (BioVision, Linda Vista Avenue, Mountain View, CA, USA). Caspase activity was determined according to the manufacturer’s protocol.

### 3.10. Data Analysis

Data were presented as mean ± standard deviation (n = 3). The statistical comparisons were performed by one-way analysis of variance (ANOVA) with Duncan’s test. The significant differences were indicated as p < 0.05 or 0.01.

## 4. Conclusions

Previous studies in our group showed that RMR extracts or red mold dioscorea (RMD) extracts fermented from *M. purpureus* NTU 568 might prevent carcinogenesis or tumor progression in animal models. We also isolated and confirmed that some azaphilone derivatives were cytotoxic to laryngeal, colon and lung cancer cell lines. In this study, a new azaphilone MPA was isolated from *M. purureus* NTU 568 fermented red mold rice, and showed moderate cytotoxicity against breast cancer cells. In conclusion, azaphilone derivatives isolated in our studies were moderately cytotoxic but tissue-specific to different cancer cell lines. These results strongly implied that fermented products from *M. purpureus* NTU 568 are potential candidates for tumor prevention due to the available amounts of azaphilone derivatives.

## Supplementary Materials

Supplementary materials can be accessed at: http://www.mdpi.com/1420-3049/17/1/664/s1.
