# Supplementary material for: Induction of Apoptosis in Human Breast Adenocarcinoma Cells MCF-7 by Monapurpyridine A, a New Azaphilone Derivative from Monascus purpureus NTU 568
Source: Molecules. 2012 Jan 11;17(1):664–73. doi: 10.3390/molecules17010664 (PMC6268682; doi:10.3390/molecules17010664)

*Molecules* **2012**, *17*, 664-673; doi:10.3390/molecules17010664

**OPEN ACCESS**

molecules

**ISSN 1420-3049**

www.mdpi.com/journal/molecules

*Article*

Induction of Apoptosis in Human Breast Adenocarcinoma Cells MCF-7 by Monapurpyridine A, a New Azaphilone Derivative from *Monascus purpureus* NTU 568

Li-Chuan Hsu ^1,2^, Ya-Wen Hsu ^1,2^, Yu-Han Liang ^1,2^, Chia-Ching Liaw ^2^, Yao-Haur Kuo ^2,3^,* and Tzu-Ming Pan ^1^,*

^1^ [Department of Biochemical Science and Technology](http://www.bst.ntu.edu.tw/BSTe.htm), National Taiwan University, Taipei 10617, Taiwan

^2^ Division of Herbal Drugs and Natural Products, National Research Institute of Chinese Medicine, Taipei 11221, Taiwan

^3^ **Graduate Institute of Integrated Medicine**, China Medical University, Taichung 40402, Taiwan

***** Authors to whom correspondence should be addressed; E-Mails: [kuoyh@nricm.edu.tw](mailto:kuoyh@nricm.edu.tw) (Y.-H.K.); [tmpan@ntu.edu.tw](mailto:tmpan@ntu.edu.tw) (T.-M.P.); Tel.: +886-2-2820-1999 ext. 7061 (Y.-H.K.);
Fax: +886-2-2823-6150 (Y.-H.K.); Tel.: +886-2-3366-4519 ext. 10 (T.-M.P.);
Fax: +886-2-3366-3838 (T.-M.P.).

Received: 21 November 2011; in revised form: 6 January 2012 / Accepted: 6 January 2012 /
Published: 11 January 2012

**Figure 1.** The ^1^H-NMR spectrum of monapurpyridine A in CDCl_3_.

**Figure 2.** The ^13^C-NMR spectrum of monapurpyridine A in CDCl_3_.


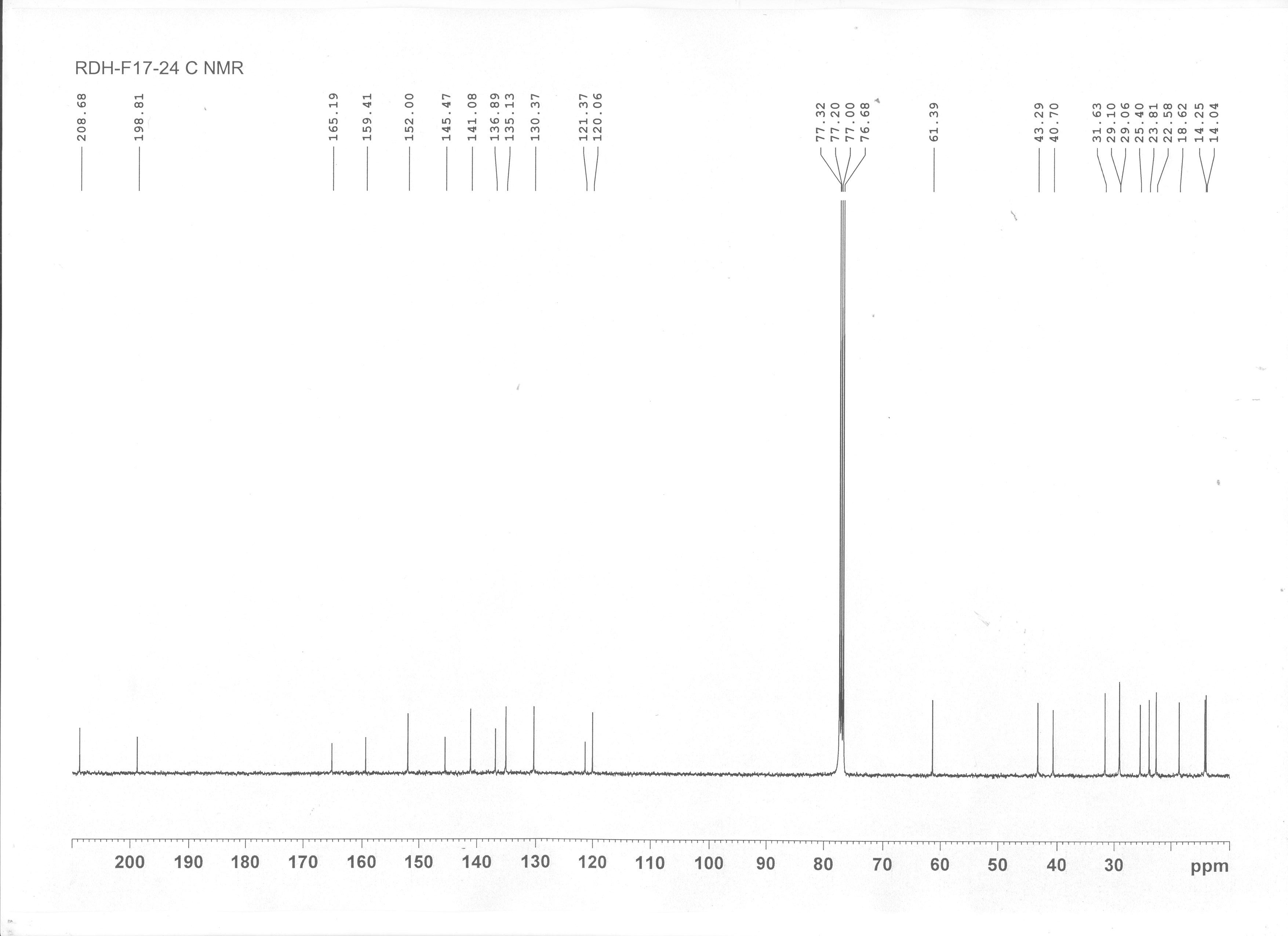

Supplement: Supplementary file 1 [file molecules-17-00664-s001.doc]
